# Comparative Microbiomes of the Respiratory Tract and Joints of Feedlot Cattle Mortalities

**DOI:** 10.3390/microorganisms10010134

**Published:** 2022-01-10

**Authors:** Chunli Li, Rahat Zaheer, Andrea Kinnear, Murray Jelinski, Tim A. McAllister

**Affiliations:** 1 Lethbridge Research and Development Centre, Agriculture and Agri-Food Canada, Lethbridge, AB T1J 4B1, Canada; lichunli_1978@hotmail.com (C.L.); rahat.zaheer@agr.gc.ca (R.Z.); 2 Large Animal Clinical Sciences, Western College of Veterinary Medicine, University of Saskatchewan, 52 Campus Drive, Saskatoon, SK S7N 5B4, Canada; andrea.kinnear@usask.ca (A.K.); murray.jelinski@usask.ca (M.J.)

**Keywords:** bovine respiratory disease, arthritis, feedlot cattle, microbiome, nasopharynx, trachea, lung, joint

## Abstract

A comparative study of microbiota of the respiratory tract and joints of bovine respiratory disease (BRD) cattle mortalities was undertaken. Nasopharynx, trachea, lung and joint samples were collected from 32 cattle that died of BRD, “cases”, and 8 that died of other causes, “controls”. Bacterial diversity was lower (*p* < 0.05) in the nasopharynx, trachea and lungs of cases as compared to controls. In cases, alpha-diversity (*p* < 0.05) was lower in the lungs and joints than the nasopharynx. *Proteobacteria*, *Tenericutes*, *Bacteroidetes*, *Firmicutes* and *Actinobacteria* were the most abundant phyla in all samples. Relative abundances of *Mycoplasma* spp. in the lung, *Pasteurella* spp. in the trachea and lung, and *Histophilus* spp. in the lung, trachea and nasopharynx of cases were higher (*p* < 0.001) than controls. *Mycoplasma* spp. comprised 20.5% of bacterial flora in the joint, 36.0% in the lung, 22.4% in the trachea and 8.8% in the nasopharynx. *Mannheimia* spp. (21.8%) and *Histophilus* spp. (10.4%) were more abundant in lungs. Cattle that died of BRD possessed less diverse respiratory microbiomes with a higher abundance of respiratory pathogens. *Mycoplasma* spp. were prominent members of pneumonic lungs and joints displaying septic arthritis.

## 1. Introduction

Bovine respiratory disease (BRD) is the most significant cause of mortalities in newly arrived feedlot calves [1], costing the North American feedlot industry over $4 billion annually due to treatment and prevention costs as well as lost productivity [2,3]. BRD is a multifactorial disease precipitated by various stressors (e.g., weaning, transportation, and commingling) that predispose cattle to viral and bacterial infections, with bacterial pathogens being considered the principle etiological agents. Primary bacterial pathogens associated with BRD include *Pasteurella multocida*, *Mannheimia haemolytica*, *Histophilus somni* and *Mycoplasma bovis* [4,5], all of which can colonize the upper respiratory tract as commensals in healthy cattle [6,7,8]. Presumably, when exposed to the appropriate combination of stressors, cattle become immunocompromised, leading to a proliferation of bacteria that colonize the lungs, resulting in pneumonia [6,9,10,11]. *M. haemolytica* (formerly *Pasteurella haemolytica)* infections in feedlot cattle are colloquially known as “Shipping fever” or “Pasteurellosis” and are characterized by a high fever and depression with postmortem findings of a severe, acute hemorrhagic fibrinonecrotic bronchopneumonia. *P. multocida* is associated with a fibrinopurulent bronchopneumonia, which results in less fibrin and necrosis and is hence less fulminant. *H. somni* is associated with purulent bronchopneumonia, severe fibrinous pleuritis, and a septicemia that may lead to secondary infections in other organs. Generally, *P. multocida*, *M. haemolytica* and *H. somni* are associated with BRD that develops within days to weeks of arrival at the feedlot. In contrast, pneumonia and polyarthritis cases associated with *M. bovis* tend to develop later in the feeding period [6,12,13,14]. In addition to colonizing the upper respiratory tract, hematologic dissemination of *M. bovis* from the lungs may lead to secondary colonization of joints and the development of polyarthritis [15].

A previous study reported that nasopharyngeal bacterial communities of cattle with BRD had lower diversity and richness as compared to their healthy counterparts [5]. Furthermore, the bacterial communities inhabiting the nasopharynx and trachea of healthy feedlot cattle were distinct from those associated with cattle suffering from bronchopneumonia [16]. Bacterial communities have also been shown to differ between the lower and upper respiratory tracts [16,17]. The microbiome of the trachea of feedlot cattle has a lower alpha diversity when compared to the nasopharynx, regardless of clinical status [16,17]. Although *M. haemolytica*, *P*. *multocida*, *H*. *somni*, and *M*. *bovis* are commonly isolated from the lower respiratory tract of pneumonic cattle, most microbiome studies have focused on the nasopharynx or trachea [5,7,8,16,18,19,20]. 

The objective of this study was to compare bacterial communities of nasopharynx, trachea, lungs and joints from cattle that had lesions consistent with bovine respiratory disease (“cases”) versus those that died of other non-BRD causes (“controls”). We hypothesized that bacterial communities would also differ by location within the respiratory system, and between the lungs and joints of cattle that died of BRD. 

## 2. Materials and Methods

### 2.1. Animals

Samples were collected from beef cattle from September 2018 to December 2019 at four commercial feedlots in Southern Alberta, Canada. On arrival, all cattle were weighed (204 to 350 kg) and received a metaphylaxis treatment of tulathromycin (Draxxin, 2.5 mg kg^−1^; Zoetis, Kalamazoo, MI, USA). Trained pen riders monitored the health of the cattle on a daily basis and removed those cattle displaying clinical signs of disease, of which BRD and lameness cases were most prevalent. Cattle were administered antimicrobials according to treatment protocols devised by the feedlot veterinarians. Tulathromycin was used as a treatment along with a variety of other antimicrobials including enrofloxacin (Baytril; Elanco Canada Ltd., Guelph, ON, Canada), ceftiofur (Ceftiocyl; Vetoquinol, Lavaltrie, QC, Excede or Excenel; Zoetis), florfenicol (Florkem; Ceva Animal Health Inc., Cambridge, ON, Canada), florfenicol/flunixin (Resflor; Merck Animal Health, Kirkland QC, Canada), oxytetracycline (Oxymycine LA 200, Oxymycine LA 300; Zoetis, Oxyvet 100 LP or Oxyvet 200 LA; Vetoquinol), and sulfadoxine/trimethoprim (Trimidox; Vetoquinol). 

Cattle (~300 head per pen) were housed in large (8500 m^2^) outdoor dirt-floor pens and were fed a balanced barley-based growing diet twice daily. Water was provided ad libitum and feed deliveries were adjusted to ensure ad libitum consumption. 

### 2.2. Sampling Procedures

Nasopharyngeal, tracheal, lung and joint samples were collected at necropsy from 32 ‘case’ cattle mortalities that died of BRD or were euthanized due to intractable septic arthritis and 8 ‘control’ cattle that died of other causes. Some individual animals exhibiting infectious disease received as many as 10 antimicrobial prior to death. Cases averaged 40 days on feed (DOF) at the time of death, with a range of 15 to 71 days. Twenty of the 32 cases had gross pathological lesions consistent with mycoplasmosis, 9 were diagnosed with chronic pneumonia polyarthritis syndrome (CPPS), 5 had necrotic bronchopneumonia, and 6 cases were either chronic pneumonia or chronic bronchopneumonia. Of the eight control cattle, seven died of accidental electrocution from a singular event. These animals were near market weight. The eighth control animal was 8 DOF when it died of bloat. A total of 132 samples were collected during this study (Table 1). Nasopharyngeal (*n* = 40), tracheal (*n* = 39), lung (*n* = 40) and joint (*n* = 13) tissues were obtained from case and control animals at necropsy by a field veterinarian Nasopharyngeal mucosal tissue was obtained by excising the nares and removing a 1–2 cm block of mucosal tissue. Deep nasal swabs were obtained using guarded nasal swabs. The trachea was transected at the mid-point between the nasal cavity and the lungs. A en bloc tracheal sample, consisting of 2–3 tracheal rings, was sectioned from the end of the transected trachea. Lung tissue, consisting of at least a 3 × 3 cm sample was excised from the boundary of the healthy and diseased lung tissue, which was most commonly in the cranioventral lung lobes. Control samples were obtained from the left cranioventral lung lobe of healthy cattle. Synovial tissue (1 × 1 cm) was excised from the joints of cases displaying septic arthritis. Only a single joint sample was obtained from a control animal. All sampling occurred under field conditions, with the ambient temperature frequently below 0 °C. Tissues were placed in individually identified plastic specimen cups. All samples were transported in a cooler by the veterinarians collecting the samples to the veterinary clinic, where they were promptly placed in a −20 °C freezer. Samples were then transferred to the laboratory and stored at −80 °C until further processing.

### 2.3. DNA Extraction, Quantification and Quality Assessment

Metagenomic DNA from samples was extracted as described by Zaheer et al. [21]. Briefly, frozen tissues (325 mg) were excised in a sterile Petri dish and transferred to a sterile 2.0 mL safe-lock snap-cap tubes containing 0.4 g of sterile zirconia beads (0.3 g of 0.1 mm and 0.1 g of 0.5 mm) and 1 mL of resuspension buffer (600 mM NaCl, 120 mM Tris-HCl, 60 mM EDTA, 200 mM guanidine isothyocynate; Fisher Scientific, Ottawa, ON). Five microliters of 1:1 ratio of β-mercaptoethanol (β-ME) and resuspension buffer were added to the sample tube, vortexed and then pre-heated (70 °C). A 10% SDS (200 μL) solution was added and the mixture was homogenized for 3 min on a OMNI bead Ruptor 9 (Omni International, Kennesaw, GA, USA) with setting = 5 M/s. The homogenate was then incubated at 70 °C for 15 min in a shaking incubator at 300 RPM. The mixture was centrifuged at 4 °C for 5 min at 16,000× *g* to obtain the supernatant. To recover DNA from any remaining intact microbes, fresh resuspension buffer (800 μL), 1:1 ratio of β-ME and resuspension buffer (5 μL) and 70 °C heated 10% SDS (200 μL) were sequentially added to the remaining pellet, mixed, homogenized and the supernatant was collected. The supernatants were further processed for DNA extraction as described by [21]. 

Nasopharyngeal swabs were suspended into enzymatic buffer (180 µL) containing mutanolysin (300 U mL^−1^) and lysozyme (20 mg mL^−1^), vortexed and then incubated at 37 °C for 1 h in a shaking incubator at 300 RPM. Proteinase K (25 µL) and 200 µL of AL buffer (without ethanol) from DNeasy Blood and Tissue kit (Qiagen, Toronto, ON, Canada) were then added, vortexed and incubated at 56 °C for 30 min. Approximately 400 mg of zircon/silica beads (0.3 g of 0.1 mm and 0.1 g of 0.5 mm) were then added and mixed using a Tissue Lyser II (Qiagen) at maximum amplitude for 3 min. The mixture was centrifuged (13,000× *g* for 5 min), and 200 µL of ethanol was added to the supernatant, followed by vortexing. From this point, the DNeasy Blood and Tissue Kit was employed as per manufacturer’s instructions. Subsequent to DNA isolation, quality and quantity of the isolated DNA was evaluated. DNA concentrations were measured by fluorescence using the Quant-iT™ PicoGreen (Thermo Fisher Scientific, Mississauga, ON, Canada). Purity of the DNA was determined by measuring the ratios of absorbance at 260/280 and 260/230 using a NanoDrop spectrophotometer (Thermo Fisher Scientific, Waltham, MA, USA). DNA preparations with a 260/280 ratio between 1.7 and 2.0 and a 260/230 ratio between 2.0 and 2.2 were regarded as suitable for further analysis. The extracted DNA was stored at −80 °C until sequenced.

### 2.4. 16S rRNA Gene Sequencing and Analysis

The 16S rRNA gene sequence libraries were generated using a two-step PCR protocol. The first PCR step amplified the V4 region of the 16S rRNA gene using the universal bacterial and archaeal primers 515-F (GTGCCAGCMGCCGCGGTAA) and 806-R (GGACTACHVGGGTWTCTAAT) [22]. The second PCR step was used to add a unique 10-bp barcode at the 5′ end of each amplicon as well as to add Illumina (Illumina, San Diego, CA, USA) adapter sequences. The 16S rRNA gene amplicons were quantified using a Quant-iT PicoGreen dsDNA assay kit (Invitrogen, Burlington, ON, Canada), pooled in equimolar ratios, and then purified with AMPure XP beads (Beckman Coulter, Mississauga, ON, Canada). Sequencing of 16S rRNA gene amplicons was carried out using an Illumina MiSeq (2 × 250; San Diego, CA, USA) and the MiSeq Reagent Kit v2 (500 cycles; Illumina) according to manufacturer’s instructions. All PCR amplification and sequencing steps were carried out at Genome Quebec (Montreal, QC, Canada).

The 16S rRNA gene sequences were processed using QIIME2 [23] and the R-package DADA2 (Version 1.40) denoise method. Briefly, the forward and reverse reads were each truncated at a length of 240 bp, quality control was performed for the reads using the QIME2, with chimeric sequences identified and removed. The reads were merged and taxonomy assigned so as to generate operational taxonomic units (OTUs) at 97% similarity, using the naïve Bayesian RDP classifier [24] and the Greengenes reference database [25]. The number of OTUs per sample and the Shannon diversity index were calculated in R using Phyloseq 1.20.0 [26] and vegan 2.4.4 [27] was used to determine the Bray–Curtis dissimilarities. Differential bacterial taxonomy of cattle that succumbed to BRD as compared to controls were identified using R-package Deseq2 [28] with Fold Change. Sequences have been submitted to NCBI under Bioproject PRJNA788973.

### 2.5. Statistical Analysis 

Nasopharynx, tracheas, lung and joint samples were randomly subsampled to 16,000 sequences, prior to the calculation of the diversity metrics and Bray–Curtis dissimilarities. Only OTUs with at least 50 reads in the samples were included in the analysis. The number of OTUs and Shannon diversity index were analyzed in R v.3.6.1 by mortality using a linear mixed model implemented with the lmer function in lme4 v 1.1.15 package [29]. The linear mixed model included the random effect of the sample site and fixed effects of BRD case or control mortalities. The number of OTUs and Shannon diversity index from BRD mortalities were analyzed in R v. 3.6.1 by tissue type using a linear mixed model implemented with the lmer function in lme4 v. 1.1.15 package [28]. The linear mixed model included the random effect of the sample site and fixed effect of tissue type. Post hoc comparisons were performed within each sample type using Tukey’s honestly significant difference test by postHoc package v.0.1.3. Microbial community structure of nasopharynx, trachea and lung samples were analyzed with vegan using permutational multivariate analysis of variance (PERMANOVA; Adonis function; 10,000 permutations) of the Bray–Curtis dissimilarities to assess the difference between BRD case and control mortalities. Similarly, microbial communities of nasopharyngeal, tracheal, lung and joint samples from BRD mortalities were analyzed with vegan using permutational multivariate analysis of variance (PERMANOVA; Adonis function; 10,000 permutations) of the Bray–Curtis dissimilarities. The differential taxonomy based on genus level was analyzed using GMPR to adjust the data, hierarchical cluster, clustering distance with Euclidean and Log fold changes for comparison. Joint sample data were not subjected to statistical analysis between BRD and control mortalities due to only having a single control sample.

## 3. Results

### 3.1. 16S rRNA Sequence Data

A total of 132 samples (Table 1) were used to assess microbiomes and sequences were filtered for size, quality, and for the presence of chimeras. A total of 7,768,164 reads (median = 59,109, minimum = 4404, and maximum = 115,795) were used to identify 4661 unique bacterial operational taxonomic units (OTUs).

**Figure 1:**
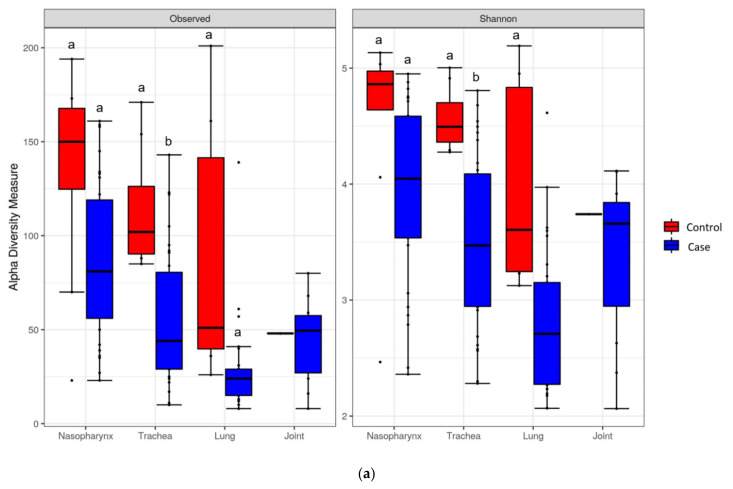

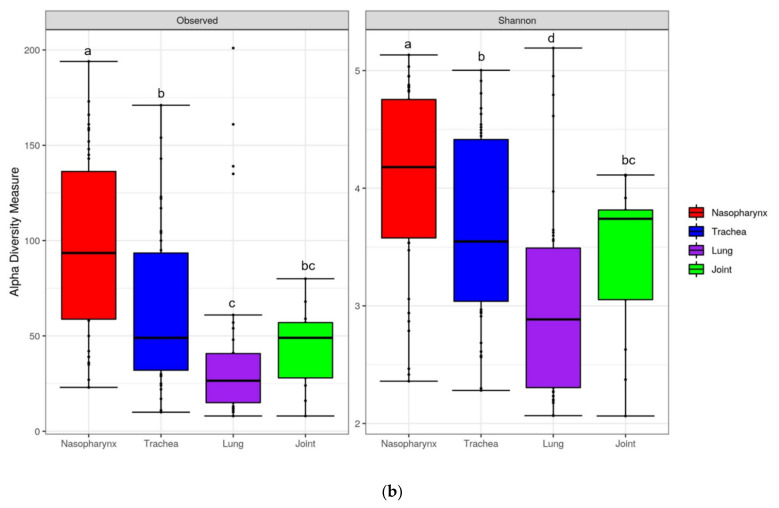
(**a**) Comparative alpha-diversity (observed and Shannon) of OTUs in respiratory tract and joint microbiota from case and control cattle. Different lowercase letters associated with each sample type differ at *p* < 0.05. (**b**) Comparative alpha-diversity (observed and Shannon) of OTUs in respiratory tract and joint microbiota from BRD mortalities (cases). Different lowercase letters differ at *p* < 0.05.

A decreasing trend in alpha diversity was observed for BRD cases as compared to controls as identified by both the number of OTUs per sample (richness) and the Shannon diversity index in all tissue types (Figure 1). A lower (*p* < 0.05) number of OTUs per sample (richness) and the Shannon diversity index were detected for both tracheal and lung samples, while there was no difference (*p* > 0.05) in the number of OTUs in nasopharyngeal samples from cases vs. controls. No statistical analysis could be conducted for the joint samples due to the availability of a single control sample (Figure 1a). Both the number of OTUs per sample and the Shannon index had similar trends among all samples (Figure 1b). The number of OTUs per sample (richness) and the Shannon index for joint samples was similar to trachea while this estimate was lower (*p* < 0.05) for the nasopharynx and higher (*p* < 0.05) for lung samples (Figure 1b). 

### 3.2. Comparative Tissue Microbiota of Cases and Controls

Permutational multivariate analysis of variance (PERMANOVA) indicated that cause of death was associated with the microbial community structure of the nasopharynx (R^2^ = 0.042; *p* = 0.003), trachea (R^2^ = 0.048; *p* = 0.005) and lungs (R^2^ = 0.063; *p* = 0.005) but not the joints. Overall, the microbiota from all three locations of the respiratory tract (nasopharynx, trachea and lung) were dissimilar between cases and controls. Similarly, PERMANOVA revealed that tissue types from cases differed in their microbial community structure between the nasopharynx and lung (R^2^ = 0.051; *p* = 0.0001), trachea and lung (R^2^ = 0.044; *p* = 0.0001), joint and nasopharynx (R^2^ = 0.032; *p* = 0.05) as well as the joint and lung (R^2^ = 0.047; *p* = 0.02). 

**Figure 2:**
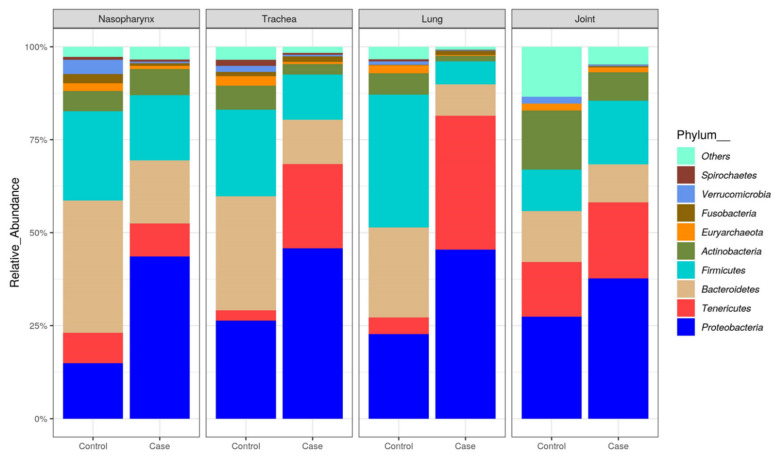
Relative abundance of bacterial 16S rRNA gene sequences at the phylum level observed in the nasopharynx, trachea and lung of cattle succumbed to BRD (cases) as compared to controls. All other classified OTUs comprising less than 1% of the total abundance are represented as others/unassigned taxa.

Twenty-nine different bacterial phyla were observed among all samples, but only *Proteobacteria*, *Tenericutes*, *Bacteroidetes*, *Firmicutes*, *Actinobacteria*, *Fusobacteria* and *Euryarchaeota* had a relative abundance greater than 1.0% (Figure 2). Five phyla represented 94.1% of the total bacterial community in both cases and controls: *Proteobacteria* (39.4%), *Tenericutes* (17.3%) *Bacteroidetes* (17.2%), *Firmicutes* (15.6%) and *Actinobacteria* (4.6%). The relative abundance of these phyla differed between cases and controls depending on the type of tissues. A predominance of *Proteobacteria* was noted in the nasopharynx (43.6%), trachea (45.8%), lung (45.4%) and joint (37.7%) of cases as compared to samples from controls, 14.9%, 26.3%, 22.7% and 27.4%, respectively. *Tenericutes*, of which *Mycoplasma* is a genus, were prevalent in all tissue types (Figure 2), comprising 20.5% of bacteria in joints as compared to 8.9% in nasopharynx, 22.7% in trachea and 36.0% in lungs. 

**Figure 3:**
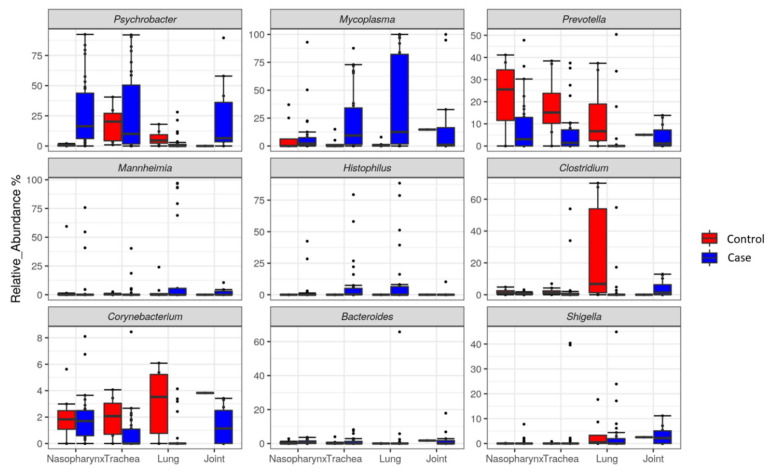
Relative abundance of top nine bacterial genera in tissue from ‘cases’ and ‘controls’. Black dots indicate outlier data points.

Both *Mycoplasma* and *Psychrobacter* were the predominant genera in samples, with a relative abundance of 17.1% and 19.9%, respectively, followed by *Prevotella* 1 (9.2%), *Mannheimia* (8.8%), *Histophilus* (4.0%), *Clostridium* (3.0%), *Corynebacterium* (1.36%), *Bacteroides* (1.3%) and *Shigella* (0.9%) in both cases and controls (Figure 3). Compared to controls, the relative abundance of *Mycoplasma* and *Psychrobacter* increased, while *Prevotella* decreased in all sample types associated with cases (Figure 3). Bacterial genera associated with cases also differed among sample types (Figure 3). For example, *Mycoplasma* was more abundant in joint, lung, trachea and nasopharynx at 20.5%, 36.0%, 22.4% and 8.8%, respectively. The relative abundance of both *Mannheimia* and *Histophilus* was 2.0 and 0.9% in joints as compared to the nasopharynx (5.7 and 2.6%), trachea (2.5 and 7.7%) and lung (21.8 and 10.4%). In addition, the relative abundance of *Prevotella* in joints was 4.1% as compared to nasopharynx (8.6%), trachea (6.2%) and lungs (3.6%). The relative abundance of *Shigella* associated with joints was 3.2% as compared to nasopharynx (0.5%), trachea (2.7%) and lungs (3.8%).

**Figure 4:**
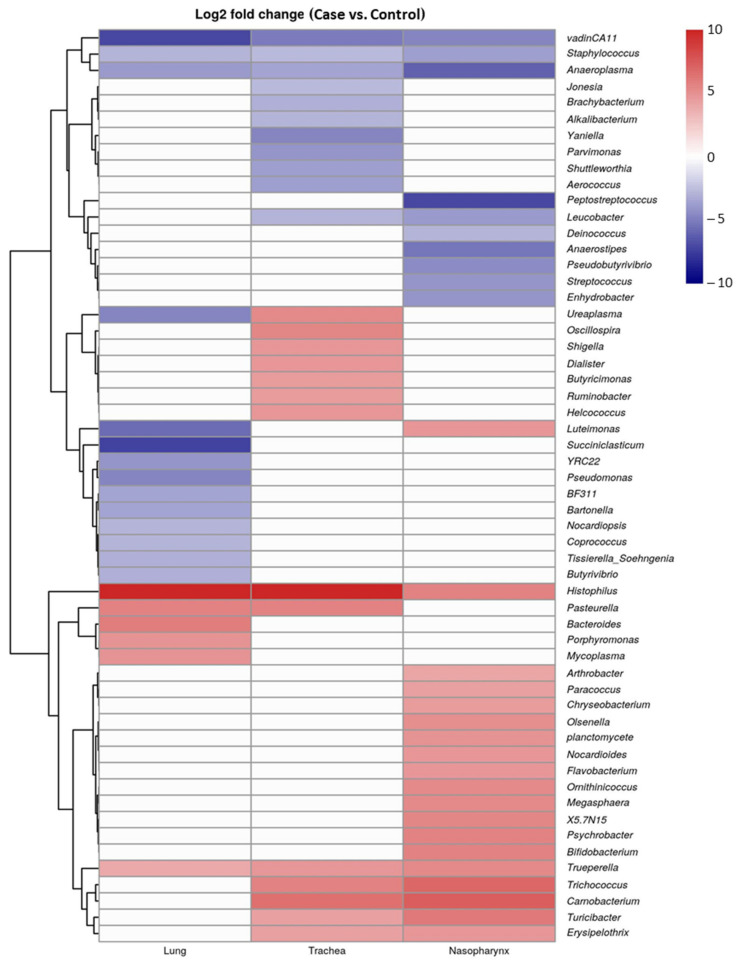
Heatmap of mean relative abundance of bacterial genera identified in respiratory tract samples from BRD cases and controls.

Differential taxonomic comparison at the genus level indicated that the bacteria linked to cases (Figure 4), including *Mycoplasma* associated with lungs, and *Pasteurella* associated with the trachea and lungs were higher than controls. *Histophilus* and *Trueperella* were higher (*p* < 0.001) in all three types of tissues from the respiratory tract of BRD cases as compared to controls. In addition, *Psychrobacter* in nasopharynx and *Bacteroides* in lungs was (*p* < 0.001) higher in cattle that died of BRD as compared to controls.

## 4. Discussion

This is the first study to characterize the microbiome of the respiratory tract and joints of feedlot cattle afflicted with arthritis and/or pneumonia. Previous microbiome studies of feedlot cattle have been limited to samples from the nasopharyngeal passages of healthy and sick animals, or the upper and lower respiratory tracts of healthy cattle [5,7,8,16,18], whereas the current study utilized tissue samples obtained at the time of postmortem examination from cattle that had either been euthanized or died naturally of nonresponsive arthritis or pneumonia. It is salient that the calves averaged 40 days on feed (DOF) at the time of death, and that all received metaphylaxis on arrival, with those displaying clinical disease being treated with antimicrobials at least once prior to death. The timing of the deaths (DOF) coupled with the animals being refractory to multiple courses of antimicrobials is highly suggestive of mycoplasmosis. In fact, five animals had overt lung lesions consistent with mycoplasmosis, while another nine animals were diagnosed with chronic pneumonia and polyarthritis syndrome (CPPS), pathognomonic for *M. bovis.* Thus, the cases in this study are representative of calves that acquired bovine respiratory disease (BRD) within days to weeks of arrival at the feedlot, with most possessing lesions that were consistent with mycoplasmosis. 

As has been previously reported, the alpha-diversity index was consistently lower for the microbiota of cases versus the controls across all tissues. The alpha-diversity in the nasopharynx of cases and controls was consistent with a previous study [30], wherein the alpha-diversity in the nasopharynx did not differ between calves diagnosed with BRD and healthy calves, regardless of age. The lower alpha-diversity in the trachea was also consistent with a study that examined steers with BRD as compared to healthy pen-mates [16]. Cases in our study also had a lower alpha-diversity of lung microbiota as compared to controls. Furthermore, cases exhibited a lower alpha-diversity in the joint and lung tissues as compared to the nasopharynx. As a diverse microbiota could provide protection against infections, a loss of diversity and shifts in the microbiota composition may establish a niche that enables virulent members of the BRD bacterial complex to proliferate and establish infections.

Previous culture-independent approaches have found differences in the nasopharyngeal and tracheal bacterial communities of healthy cattle and those diagnosed with BRD [5,16,31]. The current study detected significant differences in the composition of the microbiota in the nasopharynx as well as the tracheas and lungs of cattle that succumbed to BRD as compared to those that died of other non-pathogenic causes. The structure of bacterial communities of both the upper and lower respiratory tract were altered as a result of BRD. Our results also showed significant differences in the microbiota of the nasopharynx, trachea, lung and joint within BRD mortalities. Others also found distinct microbial communities between the nasopharynx and trachea of post-weaned Piedmontese calves or steers with or without BRD [16,17]. The fact that tracheal microbiota partly share common bacterial community members with the nasopharyngeal microbiota or lung microbiota is not surprising, as these sites are anatomically continuous. 

In agreement with our study, *Proteobacteria*, *Tenericutes*, *Bacteroidetes*, *Firmicutes* and *Actinobacteria* have been reported to be the most abundant phyla in nasopharynx and trachea of recently weaned calves and feedlot cattle [8,17,18,19,32]. The second most abundant phylum was *Tenericutes* (17.3%), which contains the class *Mollicutes* that includes *Mycoplasma*. *Tenericutes* have been reported to be the most abundant phylum in the nasopharynx and trachea of feedlot cattle comprising more than 40% of the total bacterial community [16,32]. The current study revealed a predominance of *Proteobacteria* in nasopharynx, trachea and lung, and *Tenericutes* in trachea and lungs of cattle that died of BRD as compared to controls. Furthermore, there was a predominance of *Tenericutes* in the trachea, lungs and joints, with these members being lower in the nasopharynx of cattle that died of BRD. 

The most abundant genera detected in the nasopharynx, trachea and lung of cases and controls were *Mycoplasama*, *Psychrobacter*, *Mannheimia*, *Prevotella* and *Histophilus*. This finding is similar to that observed in the nasopharynx and trachea of feedlot steers diagnosed with or without pneumonia [16]. The dominance of *Mycoplasma* in the respiratory tract of both cases and controls is in agreement with a recent study [17], which found that *Mycoplasma* represented 27.3% and 76.7% of the genera in nasopharynx and trans-tracheal samples, respectively. A greater relative abundance of *Mycoplasma* in the tracheas and lungs of cattle was not unexpected since most of the BRD cattle were euthanized because they were presumptively deemed chronic mycoplasmosis cases. Similar results were reported in tracheal samples [16] with more *M. bovis* in feedlot cattle diagnosed with bronchopneumonia or in bronchoalveolar lavage samples from necropsied feedlot cattle diagnosed with chronic suppurative pneumonia [11,14,31]. 

A number of *Mycoplasma* species including *M*. *arginini*, *M*. *bovirhinis*, *M*. *bovis*, and *M*. *dispar* have been associated with BRD in cattle, with *M. bovis* being the most abundant [31]. Furthermore, higher *Mycoplasma* abundance both in joints and lungs of cases in the present study are supportive of mycoplasmas being a causative agent of BRD and septic arthritis [15,33], which when found concurrently in the same animal is known as chronic pneumonia polyarthritis syndrome (CPPS). In an outbreak of unresponsive arthritis and pneumonia as a result of *M. bovis* infection in young calves in Jordan, lesions mediated by *M. bovis* were found in both the lungs and joints [34]. Similar findings were reported in another study where the pulmonary lesions were accompanied by polyarthritis [35].

The other most abundant genus was *Psychrobacter*, a member of the phylum *Proteobacteria*. This genus was found in the nasopharynx, trachea, lung and joint of both cases and controls. Others have also associated *Psychrobacter* with these tissues [5,16,19,30], but at low abundance and its presence in the upper respiratory tract has been inconsistent [7], with its relative abundances not differing between cases and controls. In the current study, *Psychrobacter* appeared to be enriched in the nasopharynx of cattle with pneumonia (27.4% versus 0.6% of controls). *Psychrobacters* are characteristically halotolerant and psychrophilic and have been isolated from diverse habitats including brain tissue, eye, urethra, blood and cerebrospinal fluid from humans [36], but their clinical significance, if any, is largely unknown. 

Although *Mannheimia*, *Mycoplasma*, *Histophilus* and *Pasteurella* are the most well-known bacterial pathogens associated with BRD in feedlot cattle [5,10,12,37,38,39], the results did not show an increase in relative abundance of *Mannheimia* between cases and controls. *M. haemolytica* is a commensal within the upper respiratory tract of cattle, with serotype 2 strains being less virulent in cattle than serotype 1 and 6 strains [40], a trait that cannot be distinguished via 16SrRNA sequencing. Others have found that the relative abundance of *Mannheimia* was similar in cattle that developed BRD as compared to their healthy counterparts [20]. Higher occurrence of *Pasteurella* in trachea and lung samples, and *Histophilus* in nasopharynx, trachea and lung samples from cattle with BRD as compared to controls likely reflects the important role that these genera play in this infectious disease. 

A few genera that are typically associated with the gut were also found in the respiratory tract of cattle. *Bacteroides* were found in the tracheas and lungs and were more abundant in cattle that died of BRD than in control mortalities. *Prevotella* was also found in all samples, but at similar levels of abundance between cases and controls. Others reported that the relative abundance of these genera in the nasopharynx was similar among healthy and pneumonic dairy calves [30]. More *Bacteroides* in both nasopharynx and tracheal samples from BRD feedlot cattle have been noted as compared to healthy cattle [16]. Although the potential role of these genera in the respiratory tract of cattle remains unknown, the predominance of these genera in the respiratory tract might reflect microbial seeding of this organ as a result of regurgitation of feedstuffs during rumination, eructation, or at the time of death.

In conclusion, distinct bacterial communities inhabit the respiratory tract and joints of control cattle mortalities versus the same anatomical locations of those that died of BRD or arthritis. Cases harbored less diverse respiratory tract microbiota than controls, with a higher relative abundance of BRD bacterial pathogens. Similarly, bacterial communities in the joints and lungs of cattle that died of BRD were less diverse in the nasopharynx, with a higher relative abundance of bacterial pathogens in the joints or lungs than in the nasopharynx and trachea.

## Figures and Tables

**Table 1:** Samples used to investigate comparative microbiota from two group of animals (control vs. case (BRD) mortalities).

Mortality *	Sample Types	Sample Number
Control mortalities (*n* = 8)	Joint	1 **
	Lung	8
	Nasopharynx	8
	Trachea	8
Case (BRD) mortalities (*n* = 32)	Joint	12
	Lung	32
	Nasopharynx	32
	Trachea	31

* Treatment: control, healthy cattle; case, cattle diagnosed with pneumonia. ** Only one sample for joint in the control.

## Data Availability

All Illumina sequence read data from current study have been deposited to the NCBI database as Short Read Archive (SRA) under BioProject ID: PRJNA788973.
